# Epigenetic Reprogramming Via Sodium Butyrate Induces Corneal Myofibroblast Dedifferentiation In Vitro and Inhibits Fibrosis In Vivo

**DOI:** 10.1167/iovs.66.14.59

**Published:** 2025-11-24

**Authors:** Swati Sood, Nishant R. Sinha, Anil Tiwari, Eva Ruterschmidt, Ratnakar Tripathi, Suneel Gupta, Rajiv R. Mohan

**Affiliations:** 1 Harry S. Truman Memorial Veterans’ Hospital, Columbia, Missouri, United States; 2 Departments of Veterinary Medicine & Surgery and Pathobiology and Integrative Biomedical Sciences, College of Veterinary Medicine, University of Missouri, Columbia, Missouri, United States; 3 Mason Eye Institute, School of Medicine, University of Missouri, Columbia, Missouri, United States

**Keywords:** alpha smooth muscle actin, corneal myofibroblast, dedifferentiation, corneal fibrosis, DNA methylation, HDACi, sodium butyrate

## Abstract

**Purpose:**

Abnormal corneal myofibroblast (CMF) differentiation and persistence in stroma after ocular trauma causes corneal fibrosis and impaired vision. This study tested whether epigenetic reprogramming via sodium butyrate (NaB), histone deacetylase inhibitor, could provoke CMF dedifferentiation into precursor/non-opaque corneal fibroblast/keratocyte (CSF) like cells using human in vitro and rabbit in vivo corneal fibrosis models.

**Methods:**

Healthy human cadaver corneas generated CSFs were converted into CMFs by TGFβ1 (5ng/ml) and grown in −/+ NaB (5 mM) for 72 hours. Quantitative RT-PCR and immunofluorescence measured changes in profibrotic markers (alpha smooth muscle actin [*αSMA*], collagen-III [*Col-III*], fibronectin [FN]) and fibroblast marker (fibroblast-specific protein-1 [FSP1]). Epigenetic reprogramming was measured by quantification of total HDAC activity by commercial-kit and DNA methylation using methylation-specific PCR primers for *αSMA*, *Col-III*, and *FSP1* genes. Collagen gel contraction assay (CGA) evaluated response of NaB on CMF's contractile function. In vivo clinical utility of NaB in reducing corneal fibrosis was evaluated via stereomicroscopy and Pentacam imaging in live rabbits.

**Results:**

NaB treatment to CMFs changed myofibroblast-morphology to fibroblast-like and significantly reduced *αSMA* (*P* = 0.0005), Col-III (*P* = 0.011), FN (*P* = 0.0004), and increased FSP1 (*P* *=* 0.0004) gene expression in human in vitro. Additionally, it significantly reduced total HDAC activity (*P* = 0.0009) with hypermethylation of *αSMA*, *Col-III*, and hypomethylation of *FSP1* gene promoters. Also, NaB-treated CMFs showed significantly reduced contractility (*P* < 0.0001) in CGA. Topical NaB treatment markedly reduced opacity in alkali-injured rabbit corneas in vivo.

**Conclusions:**

Dedifferentiation of CMFs via epigenetic reprogramming by NaB offers an attractive approach to treat corneal fibrosis in vivo. Additional studies are warranted.

Cornea, the outermost transparent layer of the eye, provides two thirds refraction to the eyes and is prone to infections and injury.^1^^–^^3^ A compromise in corneal clarity disrupts sharp vision. Ocular trauma activates a wound healing cascade in cornea that involves stromal fibroblasts activation to myofibroblasts, collagen synthesis, and extracellular matrix (ECM) remodeling in a feedback loop involving TGFβ1 pathway.^4^^–^^10^ Disappearance of corneal myofibroblasts (CMFs) from stroma after wound closure is essential for restoration of corneal transparency and sharp vision. The dysregulation in this orchestrated process is responsible for excessive myofibroblast formation and persistence, and irregular ECM remodeling resulting in corneal haze/fibrosis/scarring and vision impairment.^8^^–^^10^ Additionally, corneal haze/fibrosis is one of the common complications of eye surgeries, particularly those involving the corneal surface or stroma.^11^ At present, no specific effective treatment modality is available to treat or reverse corneal fibrosis. Topical anti-inflammatory drugs and corticosteroids are commonly used in patients to treat corneal inflammation and haze, but they are not sufficient to reverse corneal fibrosis/scarring.^12^^,^^13^ Recent literature reveals the ability of myofibroblasts to dedifferentiate into precursor fibroblasts in fibrotic lung, heart, liver, and kidney models.^14^^–^^18^ Furthermore, studies show that TGFβ1 mediated myofibroblast formation and dedifferentiation involve epigenetic regulation.^1^^,^^19^^–^^23^

Epigenetics refers to the inheritable changes in the phenotype involving chemical modifications in the DNA, histones or microRNA, that change chromatin structure without altering underlying DNA sequence to regulate gene expression.^24^ The most widely studied epigenetic alterations include histone modifications such as histone acetylation/deacetylation, histone methylation, and DNA methylation. These modifications change the chromatin structure, thereby altering gene expression.^24^ Growing evidence shows that epigenetic modifications like DNA methylation and histone deacetylation play a significant role in various ocular diseases, particularly those affecting the cornea.^25^^–^^29^ Acetylation of histones and non-histone proteins is governed by histone acetyltransferases and histone deacetylases (HDACs). Histone acetyltransferases transfer acetyl group from acetyl-CoA to the lysine residues on histones proteins, thereby weakening histone-DNA interactions to promote transcriptional activation. HDACs remove the acetyl groups from acetylated histones that lead to chromatin condensation and suppression of gene transcription.^24^^,^^25^ DNA methyltransferases (DNMTs) facilitate DNA methylation by transfer of methyl group from S-adenosyl methionine to the fifth carbon of cytosine to form 5-methylcytosine (5mC) in CpG islands. In mammals, four isoforms of DNMTs are reported namely, DNMT1, DNMT 3a, DNMT 3b and DNMT 3L. Although DNMT1 maintains established methylation pattern in the cell during replication, DNMT3a and DNMT3b are essential for establishing de novo methylation during development, and DNMT3L is reported to have low enzyme activity. DNA hypermethylation at gene promoters and histone deacetylation are responsible for gene repression whereas DNA hypomethylation is associated with gene activation.^24^^,^^25^

Studies show the involvement of HDACs in tissue fibrogenesis.^30^^–^^35^ In humans, total 18 HDACs are identified that are classified into four classes: Class I includes HDACs 1, 2, 3, and 8; Class II includes HDACs 4, 5, 6, 7, 9, and 10; Class III, also known as sirtuins (Sirt) include Sirts 1–7; and Class IV, which includes HDAC11. HDAC2 is reported to be associated with cardiac fibrosis whereas HDAC4 is linked to fibroblast trans-differentiation to myofibroblast in skin fibrogenesis.^20^ Epigenetic drugs targeting histone modifications particularly histone acetylation, have shown promise in treating fibrosis in non-ocular tissues including lung, kidney, skin, liver; however, limited literature is available regarding corneal fibrosis.^34^^–^^39^ HDAC inhibitors (HDACi) are the molecules that bind to HDACs to block their functions. HDACi regulate gene expression by preventing HDACs from removing of acetyl group from histones and non-histone proteins which influence variety of cellular processes like cell cycle progression, cellular differentiation, and cell death.^30^ The present study investigated the effects of sodium butyrate (NaB), a natural HDACi, on the dedifferentiation of corneal myofibroblasts and explored the underlying mechanism using an established human in vitro model of corneal fibrosis. Further, we explored the clinical applicability of topical NaB in treating corneal fibrosis in vivo using a standard alkali-injury rabbit model.

Sodium butyrate, a short chain fatty acid, is one of the effective epigenetic modulators that influence gene expression by inhibiting HDACs to either stimulate or repress the expression of specific genes. It is a broad-spectrum HDACi, targeting both class I and II HDACs.^40^^–^^42^ Sodium butyrate is reported bind to the zinc sites within HDAC enzyme thereby preventing histone deacetylation to regulate gene expression.^43^ Studies reveal that butyrate is effective in inhibiting HDAC activity leading to histone hyperacetylation and activation of genes that control cell cycle progression, thereby arresting cell proliferation. Further, HDACi are shown to modulate DNA methylation to regulate gene expression.^43^^–^^46^ Fibroblasts and myofibroblasts are characterized by distinct morphology, ECM production, deposition, TGF-β1 activation, and chemotaxis.^2^^–^^5^^,^^47^ This study assessed a novel hypothesis that myofibroblast can be dedifferentiated via epigenetic modulation by HDACi. NaB treatment to cornea induces myofibroblast dedifferentiation via epigenetic reprogramming by regulating histone acetylation and DNA gene promoter methylation of fibrotic genes in cornea locally and offers an innovative approach to alleviate corneal fibrosis in vivo.

## Methods

### Human Corneal Stromal Fibroblast and Corneal Myofibroblast Cultures

Primary fibroblast cultures were established using corneas from deceased human donors, sourced from Saving Sight in Kansas City, MO, USA, following a previously published protocol.^48^ All cultures were established and expanded in Minimum Essential Media (MEM) supplemented with 10% FBS, 100 U/ml of penicillin, and 100 mg/ml of streptomycin and amphotericin B (0.25 mg/ml), vitamins, HEPES, ciprofloxin (Thermo Fisher Scientific, Waltham, MA, USA) at 37°C in 5% CO_2_. To generate corneal myofibroblasts (CMFs), CSFs were stimulated with TGFβ1 (5 ng/µL, PeproTech; Thermo Fisher Scientific) for 72 hours in serum free conditions following our laboratory standard protocol. All in vitro experiments were performed at least three times for reproducibility and validity of results.

### NaB Dosing and Cellular Viability Assay

MTT (3-[4,5-dimethylthiazol-2-yl]-2,5-diphenyltetrazolium bromide) assay was used to evaluate the effect of NaB on the viability of CMFs (Thermo Fisher Scientific). In this method, metabolically active living cells with active mitochondrial dehydrogenases convert MTT, a tetrazolium compound, into purple formazan precipitates. The total amount of color produced can be measured by spectrophotometry which is directly proportional to the number of viable cells. Briefly, 7 × 10^3^ CMFs were seeded in each well of a 96-well culture plate and incubated for 24 hours at 37°C in humidified 5% CO_2_ incubator. The cells were treated with NaB in a dose- and time-dependent manner (0.1–20 µM for 24, 48, and 72 hours). Thereafter, 20 µL of MTT (5 mg/mL in PBS) was added to each well and incubated for four hours at 37°C in humidified 5% CO_2_ incubator. Medium containing MTT was removed and 100 µL of dimethyl sulfoxide was added to each well followed by gentle shaking for 10 minutes. The absorbance at 570 nm was measured spectrophotometrically (BioTek Synergy H1 Multimode Reader; Agilent Technologies Inc., CA, USA). A dose of 5 mM NaB for 72 hours was most tolerable for CMFs and chosen to carry out all subsequent experiments. All culture experiments were repeated thrice for reproducibility. CSFs served as baseline control for the experiments comparing effects of NaB on CMFs.

### Genomic DNA and Total mRNA Isolation

Genomic DNA and total mRNA were extracted from CSFs and CMFs using all prep DNA/RNA mini kit (Cat no. 80204; Qiagen, Germantown, MD, USA). The DNA/RNA purity was determined by measuring the ratio of absorbance at A260/A280 using NanoDrop spectrophotometer (Thermo Fisher Scientific). A ratio of 1.8/2.0 was taken as acceptable value for purity of DNA and RNA, respectively.

### cDNA Synthesis and Quantification of Gene Transcript Expression by Quantitative Real-Time PCR

Total mRNA (500 ng) was converted to cDNA using a commercial kit with Avian Myeloblastosis Virus (AMV) Reverse Transcriptase enzyme (Promega, Madison, WI, USA) following manufacturer's instruction. Quantitative real-time PCR (qRT-PCR) was performed to measure the expression levels of various genes in tested conditions using standard amplification program comprising 95°C for 10 minutes (initial denaturation) followed by 40 cycles of denaturation for 15 seconds at 95°C, primer annealing at 60°C for 60 seconds using the QuantStudio6 Real-Time PCR System (Applied Biosystems, Carlsbad, CA, USA). Each qRT-PCR reaction (20 µL) contained 10 µL of 2X SYBR green mastermix (Applied Biosystems), 200 nM of forward primer and reverse primer (1 µL) and 50 ng (2–4 µL) of cDNA. The forward and reverse primers sequences for the genes *αSMA* (alpha smooth muscle actin), *Col III* (collagen type-III), *FN* (fibronectin), *FSP1* (fibroblast-specific protein 1), and *EEF1A1* (eukaryotic translation elongation factor 1 alpha 1; housekeeping gene) are enlisted in Table 1. The difference in normalized Ct value (2^−ΔΔCt^) was used to quantify relative fold-change in gene expression between various groups/samples. *EEF1A1* was used as a reference gene to normalize the data. qRT-PCR was performed on samples obtained from three independent experiments with two technical replicates for each condition and mean fold change in mRNA expression were reported.

**Table 1.** List of Primers Used for Gene Expression Analysis

Gene Name	Forward	Reverse
*EEF1A1*	5′- CTT TGG GTC GCT TTG CTG TT -3’	5'- CCG TTC TTC CAC CAC TGA TT -3′
*αSMA*	5′- TGG GTG ACG AAG CAC AGA GC -3′	5′- CTT CAG GGG CAA CAC GAA GC -3′
*Col III*	5′- CCACTTGGGATTGCTGGGAT -3′	5′- CTT CCC CTA GGA CCT GGC AT -3′
*FN*	5′- CCC AAC TGG CAT TGA CTT TT -3′	5′- CTC GAG GTC TCC CAC TGA AG -3′
*FSP1*	5′- CAG AAC TAA AGG AGC TGC TGA CC -3′	5′- CTT GGA AGT CCA CCT CGT TGT C -3′

### Immunostaining and Fluorescent Microscopy

Immunofluorescence (IF) studies were conducted in CSFs and CMFs grown in 24 well plates. After an initial rinse with 1× sterile PBS, cells were fixed with 4% paraformaldehyde (PFA; 100 µL) for 20 minutes at room temperature (RT). Cells were washed thrice with 1× PBS (for five minutes each) followed by blocking with 5% BSA in PBST (1× PBS + 0.05% Tween20) for one hour at RT. Cells were incubated with the primary antibodies in 5% BSA-PBST overnight at 4°C. After three washings with 1× PBS (five minutes each), the cells were incubated with appropriate labeled secondary antibodies diluted in 2.5% BSA-PBST for one hour at RT. The cells were washed thrice with 1× PBS (five minutes each) and nuclei were counterstained with VECTASHIELD antifade mounting medium with DAPI (Cat no. H-1200; Vector Lab, Newark, NJ, USA) to visualize the nuclei. The primary antibodies were monoclonal anti-human αSMA (1:250, Cat no. M085101-2; Aligent Technologies, Santa Clara, CA, USA) and FSP1 (1:250, Cat no. AB124805; Abcam, Cambridge, MA, USA). The secondary antibodies were Fluor-488 labeled goat anti-mouse (1:250 in 2.5% BSA in PBST, Cat no. A11029; Invitrogen, Carlsbad, CA, USA) for *αSMA* and Alexa Fluor-594 labeled donkey anti-rabbit antibody (1:250 in 2.5% BSA in PBST, Cat no. A21207; Invitrogen) for FSP1. Images of IF studies were captured using a fluorescent microscope (Leica DMI 4000 B; Leica Microsystems, Wetzlar, Germany). The αSMA stress fiber and FSP1 protein quantitation was performed with ImageJ software, and results are reported as normalized mean fluorescence intensity (MFI) ± SEM. The change in MFI was calculated and expressed in percentage.

### Preparation of Nuclear Extract and Evaluation of HDAC Activity in Human Cornea In Vitro

Nuclear protein was isolated from CSFs and CMFs (−/+ NaB) in vitro using a commercial kit (Cat no. 289882; Abcam) following manufacturer's instructions. The protein concentration was determined by the bicinchoninic acid assay, according to the manufacturer's protocol (Thermo Fisher Scientific). The change in total HDAC enzyme activity was quantified in the nuclear extract with Epigenase HDAC Activity/Inhibition Direct Assay Kit (Colorimetric; Cat no. P-4034; EpigenTek, Farmingdale, NY, USA) following manufacturer's instructions. This kit allows measurement of both HDAC activity, as well as inhibition in the presence of HDACi with protein ranging from 0.5 to 20 µg. Nuclear extract (10 µg) was used to evaluate inhibition caused by NaB. Percent HDAC inhibition was calculated using the formula: Inhibition % = (1 – Inhibitor Sample OD – Blank OD)/No Inhibitor Sample OD – Blank OD × 100%, where Inhibitor sample = CMF + NaB; No inhibitor sample = CMF − NaB; and Blank = no cell. For representation purposes, the results of HDAC inhibition by NaB are presented as percent total HDAC activity considering non-NaB-treated CMFs as 100% HDAC activity.

### DNA Gene Promoter Methylation Analysis by Methylation-Specific PCR

Briefly, genomic DNA (500 ng) was modified with bisulfite using EZ DNA Methylation-Gold Kit (Cat no. D5005; Zymo Research, Irvine, CA, USA) following the manufacturer's protocol. DNA gene promoter methylation of *αSMA*, *Col III*, *FSP1* genes was assessed by methylation specific-PCR (MSP) using a primer sets specific for methylated and unmethylated promoter sequence (Table 2). The primers were designed using MethPrimer database (http://www.urogene.org/cgi-bin/methprimer/methprimer.cgi). Each PCR reaction included buffer, MgCl_2_, forward and reverse primers (0.2 µM each), hot-start Taq DNA polymerase (1 unit, Promega), and 0.25 mM of dNTP (cocktail of dATP, dGTP, dCTP, and dTTP) and 40 ng bisulfite modified DNA. PCR amplification was carried out using the thermal cycler (Bio-Rad, Hercules, CA, USA) by following a program of initial denaturation at 95°C for 10 minutes, followed by 40 cycles of denaturation at 95°C for 30 seconds, annealing at primer specific temperature for 30 seconds, and extension at 72°C for 30 seconds, followed by final extension for eight minutes at 72°C. *ACTB* (β-actin), a housekeeping gene, was used as reference control and *DAPK* (death-associated protein kinase) gene served as an internal control for bisulfite conversion. The results of MSP were interpreted based on the band with methylated (M) primer versus unmethylated (UM) primer. An unmethylated sample will show a band only with the UM primer, a methylated sample will show a band only with the M primer, and a partially methylated will show bands with both primers (M and UM). A stronger band in the methylated lane relative to the unmethylated lane indicates a higher proportion of methylated DNA (hypermethylation), whereas the presence of a band in the unmethylated lane signifies the presence of unmethylated DNA.

**Table 2.** List of Primers Used for Methylation Analysis

Gene Name	Forward	Reverse	Amplicon
*αSMA* (M)	5′- TTT GGA TAT TAG ATG TAA TTA GCG A -3′	5′- CAA CAA AAT ACC AAA CAT AAC GAA -3′	237bp
*αSMA* (UM)	5′- GTA ATT TGG ATA TTA GAT GTA ATT AGT GA -3′	5′- CCA ACA AAA TAC CAA ACA TAA CAA A -3′	236bp
*Col III* (M)	5′- GGA TAT TAA GAT TAT TTT GAT TAA TAC GG -3′	5′- TCC CGA ATA ACT AAA ACT ACA AAC G -3′	107bp
*Col III* (UM)	5′- GGA TAT TAA GAT TAT TTT GAT TAA TAT GG -3′	5′- CTC CCA AAT AAC TAA AAC TAC AAA CAC -3′	107bp
*FSP1* (M)	5′- TAT GTT TGT TGG GTT GCG TAT TC -3′	5′- CTA CAA AAA CCA TAC ACC TTC TCG A -3′	144bp
*FSP1* UM	5′- TTT ATG TTT GTT GGG TTG TGT ATT T -3′	5′- ACA AAA ACC ATA CAC CTT CTC AAA -3′	146bp
*ACTB*	5′- TGG TGA TGG AGA GGT TTA GTA AGT -3′	5′- AAC CAA ATA AAA CCT ACT CCT CCC TTA A -3′	132bp

ACTB, beta-actin.

### Collagen Gel Contraction Assay and In-Gel Immunofluorescence

Collagen gel contraction assay (CGA) was performed following a previously reported protocol.^49^ Briefly, 400 µL of 1% BSA in PBS was added to each well of a 24-well plate followed by incubation at 37°C overnight, to coat the wells before performing the CGA. Cells (CSFs, NaB-untreated and -treated CMFs) grown in six-well plates were trypsinized using TripLE (cat no. 12604013; Gibco, Thermo Fisher Scientific) and washed twice with media without serum. 50,000 cells [CSF/CMF (−/+NaB)] were mixed with neutralized collagen solution (1×) prepared by mixing collagen type I (cat no. A1048301; Gibco, Thermo Fisher Scientific) with MEM media (10×) containing HEPES (1M), NaHCO_3_ (7.5%), and NaOH (10 M) to a final concentration of 0.5 and 1 mg/mL. Before performing the CGA, BSA was aspirated, and 200 µL of the cell-collagen mix was added to each of the precoated wells. The collagen gels were incubated for 90 minutes to permit gelation. For the negative control, the collagen mix without cells were added to the wells. After solidification, the gels were dislodged from the edges with the help of 10 µL tip and serum free media (600 µL) was carefully added (with slight force) to float the gels. The gel contraction was monitored in a time-dependent manner (0–72 hours), and the extent of contraction was determined by measuring the surface area of the gel using ImageJ software. Contraction in the negative control (no cell) set was taken to be 0%. The gel images were taken using iBright 1500 imaging system (Thermo Fisher Scientific). Results of CGA are presented as percent gel contraction relative to negative control.

In-gel IF was carried out using standard methods to evaluate dedifferentiation effect of NaB on CMFs at a functional level. The cells encapsulated collagen gels were fixed with 4% PFA at RT for one hour in the 24-well plate followed by three washings of 10 minutes each with sterile 1× PBS (300 µL). The collagen gels were permeabilized with PBST (200–300 µL) containing 0.5% Triton X-100 for 20 minutes and blocked in 10% BSA for one hour. The gels were incubated with unlabeled primary anti-human αSMA antibody diluted in PBST (0.05% Tween-20/PBS; 1:100; Cat no. M085101; Aligent Technologies) at 4°C overnight. After three washing with 1× PBS for 10 minutes each, the gels were incubated with Alexa Fluor-594 labeled anti-αSMA secondary antibody (1:250 in PBST, Cat # A21135, Invitrogen, USA) for 1 h at room temperature. Gels were washed three times with 1× PBS for 10 min each, after removing secondary antibody and counterstained with DAPI (1 mg/ml, 1:100, Cat no. H-1200; Vector Lab, USA) for 15 minutes to stain the nuclei. For phalloidin staining, following blocking with 10% BSA and washing steps, gels were stained with labeled phalloidin (F-actin) antibody with Alexa flour-488 (1:200 in PBST, Cat no. A12379; Invitrogen) for 20 minutes at RT. Gels were washed by three time (10 minutes each) with 1× PBS to remove excess antibody and nuclei were counterstained with DAPI for 15 min. Images were captured using a fluorescent microscope (Leica DMI 4000 B; Leica). The αSMA stress fiber quantitation was performed with ImageJ software and results are reported as normalized MFI ± SEM and the change in MFI was calculated in percentage.

### In Vivo Rabbit Studies

All animal experiments were conducted in compliance with the approved Animal Care and Use Committee (ACUC) protocol of the University of Missouri, Columbia, MO, USA. Female New Zealand white rabbits (1.6–2.0 kg) procured from the Charles River Lab Inc., Wilmington, MA, USA, were housed in animal facility under standard conditions specified by the ACUC, University of Missouri. Animal handling and treatment conformed to the ARVO Statement for the Use of Animals in Ophthalmic and Vision Research.

A total of 12 rabbits were used for the study, and all procedures were carried out under general anesthesia, administered via intramuscular injection of ketamine hydrochloride (50 mg/kg) and xylazine hydrochloride (10 mg/kg). Topical ophthalmic anesthetic, proparacaine hydrochloride (0.5%, two drops), was applied onto the eye to minimize pain and discomfort to animals, prior to the alkali injury and clinical eye examination and imaging. To induce alkali injury, 8 mm filter disc soaked in 0.5 N NaOH was applied topically covering the central cornea for 30 seconds (one time only) followed by flushing with 20 mL BSA to get rid of excess alkali. Unilateral alkali injury/treatment was induced in each animal, whereas the uninjured contralateral eye served as naïve control. This research design was followed in accordance with the ACUC approved 3Rs framework of Replacement, Reduction, and Refinement to minimize unnecessary use of animals in research. Rabbits were thermally supported during the anesthetic recovery period. Rabbits were divided into three groups; each group contained four rabbits. Group 1: rabbits with alkali injury alone; Group 2: alkali injury followed by topical NaB (5 mM, two drops ∼50 µL) one day after injury twice daily for seven days; and Group 3: alkali-injury followed by topical NaB (5 mM, two drops) treatment started seven days after injury (allowing pathology to develop) twice daily for seven days, and naïve control group (the contralateral uninjured corneas). Corneal tissues were collected after animals were humanely euthanized under general anesthesia via intravenous administration of Beuthanasia-D (150 mg/kg; Schering-Plough Animal Health, Union, NJ, USA) according to our published method.^2^ Each cornea was cut in half; one half was rapidly frozen in liquid nitrogen for storage at −80°C for molecular analysis, and the other half was embedded in OCT compound (optical cutting temperature; Sakura FineTek, Torrance, CA, USA) in 24 × 24 × 5 mm molds (Thermo Fisher Scientific) to form tissue blocks for storage at −80°C, following the previously described method.^2^

### Multimodal Biomicroscopic Corneal Imaging and Clinical Eye Examination in Live Rabbits

In live animals, status of overall ocular health and corneal transparency in treated and untreated eyes of four groups was evaluated clinically with a slit-lamp microscope (SL-15; Kowa Optimed Inc., Torrance, CA, USA) and multimodal corneal imaging with stereomicroscope (MZ16F; Leica Microsystems) and Pentacam HR imaging system (Oculus, Wetzlar, Germany) periodically. To prevent dryness, irritation, and burning during the procedures, eyes were hydrated with artificial tears. The corneal haze was graded using the Fantes scale, by at least two independent observers (SS, NRS, SG, or RT), masked to the treatment group, as reported previously.^50^ Corneal imaging with Oculus Pentacam HR imaging system (Oculus) provides precise information on the depth and degree of corneal opacity on a three-dimensional corneas map in live rabbit in a color-coded manner.^51^ The rotating Scheimpflug camera generates three-dimensional images revealing information on corneal densitometry, curvature, and thickness at different regions of the cornea. The corneal images were processed and analyzed with built-in V1.21r65 software in the machine. Corneal opacity was measured in terms of total average corneal densitometry of the corneal layers from 0–12 mm thickness.

### Statistical Analysis

Data analyses were carried out using GraphPad Prism 9.2 software (GraphPad Software, La Jolla, CA, USA). Depending on the experimental design, either Student's *t*-test with Welch correction (for two groups) or one-way ANOVA with a post hoc test (Tukey's or Bonferroni's) for multiple comparisons, was performed to determine differences between the groups. A *P* < 0.05 was considered significant. All results are reported as mean ± SEM.

## Results

### NaB Changes Myofibroblast Morphology and Modulates Expression of Fibrotic Genes at Protein Levels In Vitro

**Figure 1.**
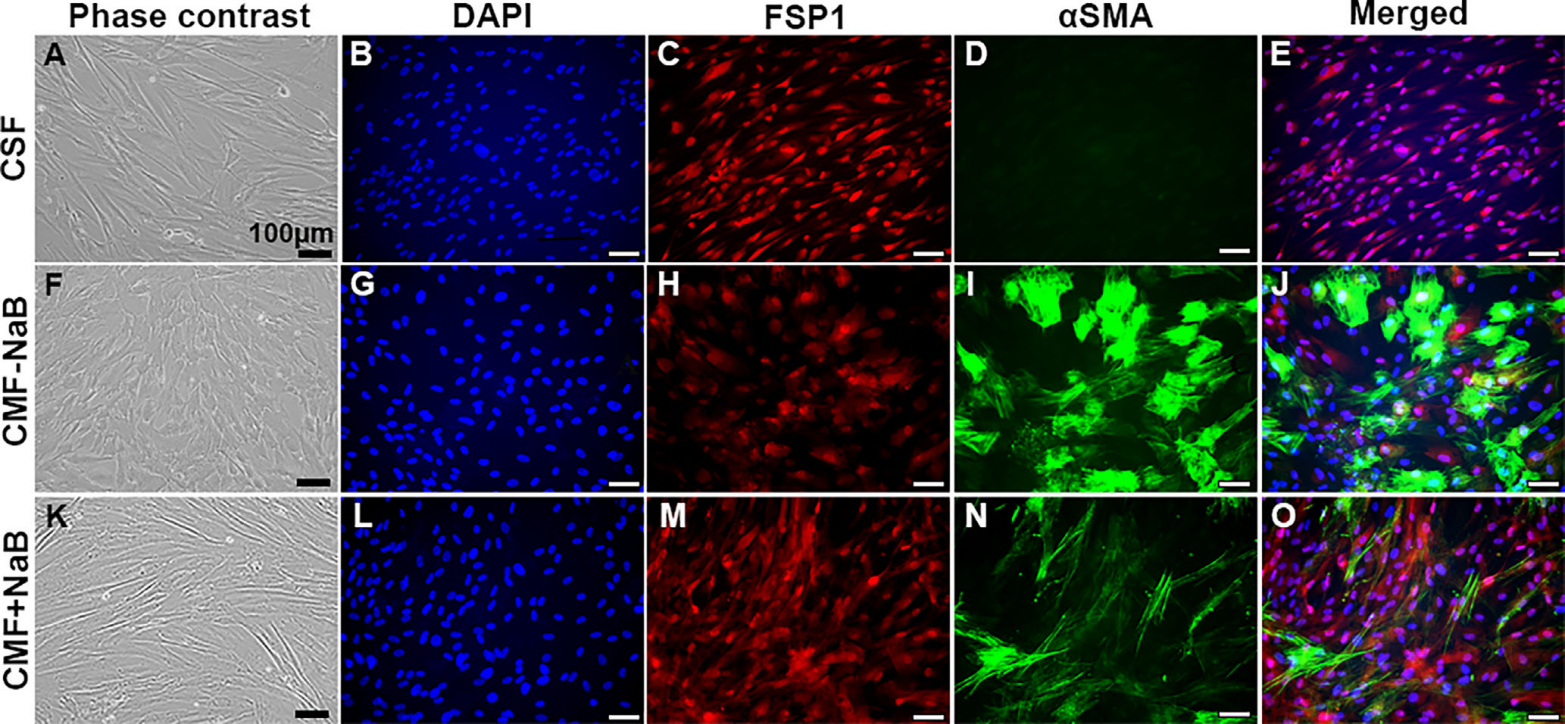
Representative phase contrast and immunofluorescence images showing changes in the cellular morphology and expression of fibrotic gene (FSP1 and αSMA) at protein levels in myofibroblast [CMF; CSF+TGFβ1 (5 ng/ml, 72 h in serum free conditions)] cells in presence or absence of sodium butyrate (NaB) compared to fibroblast (CSF grown in serum free conditions for 72 h; **A**–**E**) cells. NaB treatment allowed CMFs to acquire fibroblast like phenotype (**K**) compared to CMF-NaB (**F**). CMF (**G–J**) showed a low the levels of FSP1 (H & J; 1.26 ± 0.08, *p* = 0.11) and a significantly high αSMA levels (I & J; 3.18 ± 0.12, *p* < 0.0001) levels compared to CSFs (C, 2.37 ± 0.43 & D, 0.21 ± 0.11, respectively), while NaB treatment of CMFs (**L**–**O**) showed a significantly reduced expression of αSMA (N & O; 1.16 ± 0.1, *p* < 0.0001), and a parallel gain of FSP1 (M & O; 2.13 ± 0.26, *p* = 0.24) protein expression compared to untreated CMFs (**G–J**). αSMA, alpha smooth muscle actin; CMF, corneal myofibroblast; CSF, corneal stromal fibroblast; FSP1, fibroblast specific protein 1.

Figure 1 shows the cellular morphology and the expression of myofibroblast (*αSMA*, alpha smooth muscle actin) and fibroblast (*FSP1*, fibroblast specific protein 1) marker genes in CSFs and CMFs treated with or without NaB. The CSFs demonstrated spindle-shaped, thin, and elongated fibroblastic morphology (Fig. 1A) while CMFs showed typical flattened phenotype (Fig. 1F). The NaB treated CMF showed transformation of myofibroblastic morphology towards fibroblastic morphology under phase contrast microscope (Fig. 1K). The changes in the myofibroblast to fibroblast-like morphology were verified via double immunofluorescence (IF) using antibodies specific for αSMA and FSP1. As evident from Figure 1, NaB-treated CMFs (Figs. 1K–O) showed not only reduced stress fiber-laden myofibroblast indicator, αSMA (1.16 ± 0.1, *P* < 0.0001; Fig. 1N) but also had increased expression of fibroblast indicator, FSP1 (2.13 ± 0.26, *P* = 0.24; Fig. 1M) at 72 hours, which was contrary to the NaB-untreated CMF (Figs. 1F–J) that showed low FSP1 (1.26 ± 0.08, *P* = 0.11; Fig. 1H) and high αSMA (3.18 ± 0.12, *P* < 0.0001; Fig. 1I) expression. Nearly 63.5% of NaB-treated CMFs revealed diminished myofibroblast (Fig. 1J vs. 1O). The pattern of FSP1 protein expression in NaB-treated CMFs (2.13 ± 0.26; Figs. 1L–O) was comparable to its expression observed in the baseline CSFs (2.37 ± 0.43; Figs. 1B–E) in IF analysis.

### NaB Changes Gene Transcript Expression of Myofibroblast and Fibroblast Specific Genes

**Figure 2.**
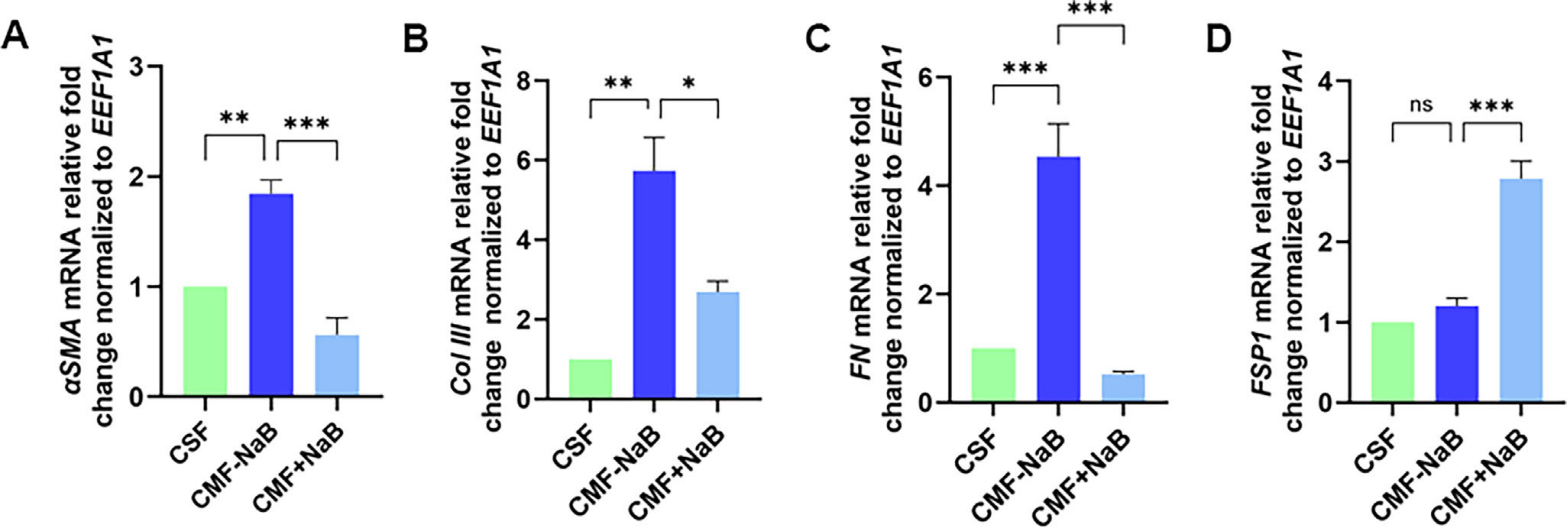
Bar graphs showing relative fold change at gene transcript levels of fibrotic: **(A)** *αSMA*, **(B)** *Col III*, **(C)** *FN*, and fibroblast: **(D)** *FSP1* genes as quantified by qRT-PCR in CMF [CSF+TGFβ1 (5 ng/mL, 72 hours in serum-free conditions)]^−/+^ NaB (5mM, 72h) compared to CSF (serum free conditions for 72h). *EEF1A1* gene was taken as housekeeping gene for relative quantification. NaB treatment of CMFs showed significant reduction of *αSMA* (*p <* 0.001), *Col III p <* 0.05), *FN* (*p <* 0.001) and a significant increase in *FSP1* (*p <* 0.001) gene expression. *αSMA*, alpha smooth muscle actin; CMF, corneal myofibroblast; *Col III*, collagen type III; CSF, corneal stromal fibroblast; *EEF1A1*, eukaryotic translation elongation factor 1 alpha 1; *FN*, fibronectin; *FSP1*, fibroblast specific protein 1.

Figure 2 reveals loss of myofibroblast marker (*αSMA*, *Col III*, and *FN*) and gain of fibroblast marker (*FSP1*) at gene transcripts levels in NaB-treated CMFs. A significant decrease in mRNA levels of *αSMA* (two fold, *P* = 0.0005), *Col III* (four fold, *P* = 0.01), and *FN* (three fold; *P* = 0.0004) and gain of fibroblast-marker (*FSP1*, two fold; *P* = 0.0004) was noted in NaB-treated CMFs compared to NaB-untreated CMFs. As expected, NaB-untreated CMFs demonstrated significantly increased myofibroblast markers (*αSMA*, *P* = 0.004; *Col III*, *P* = 0.001; and *FN*, *P* = 0.0008) and nonsignificant fibroblast expression (*FSP1*; ns) compared to the baseline CSFs.

### NaB Treatment Inhibits HDAC Activity In Vitro

**Figure 3.**
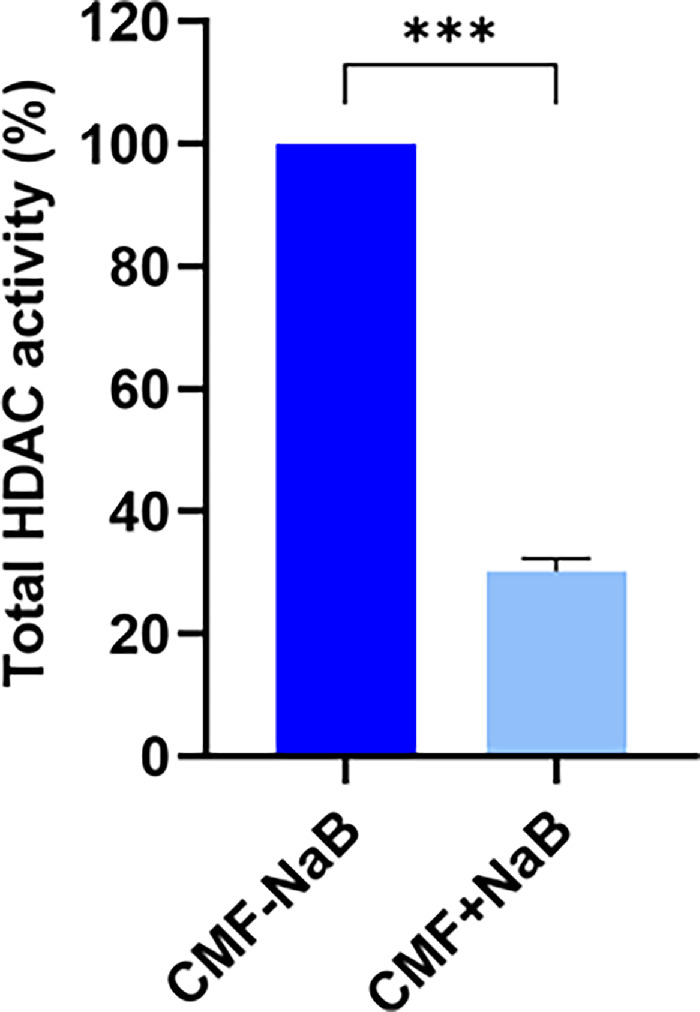
Bar graph showing effect of NaB on total HDAC activity on CMFs. NaB significantly inhibited total HDAC activity by 69.77% (*p =* 0.0009) with total HDAC activity of 30.23% ± 2.1% considering 100% activity in CMF without NaB treatment.

Figure 3 demonstrates a significantly reduced total percent HDAC activity in NaB-treated CMFs (30.23% ± 2.1%; *P* = 0.0009) compared to the NaB-untreated CMFs. This data indicated that NaB-induced dedifferentiation of CMF involves epigenetic regulation.

### NaB Treatment Modulates DNA Promoter Methylation of Fibrotic Genes

**Figure 4.**
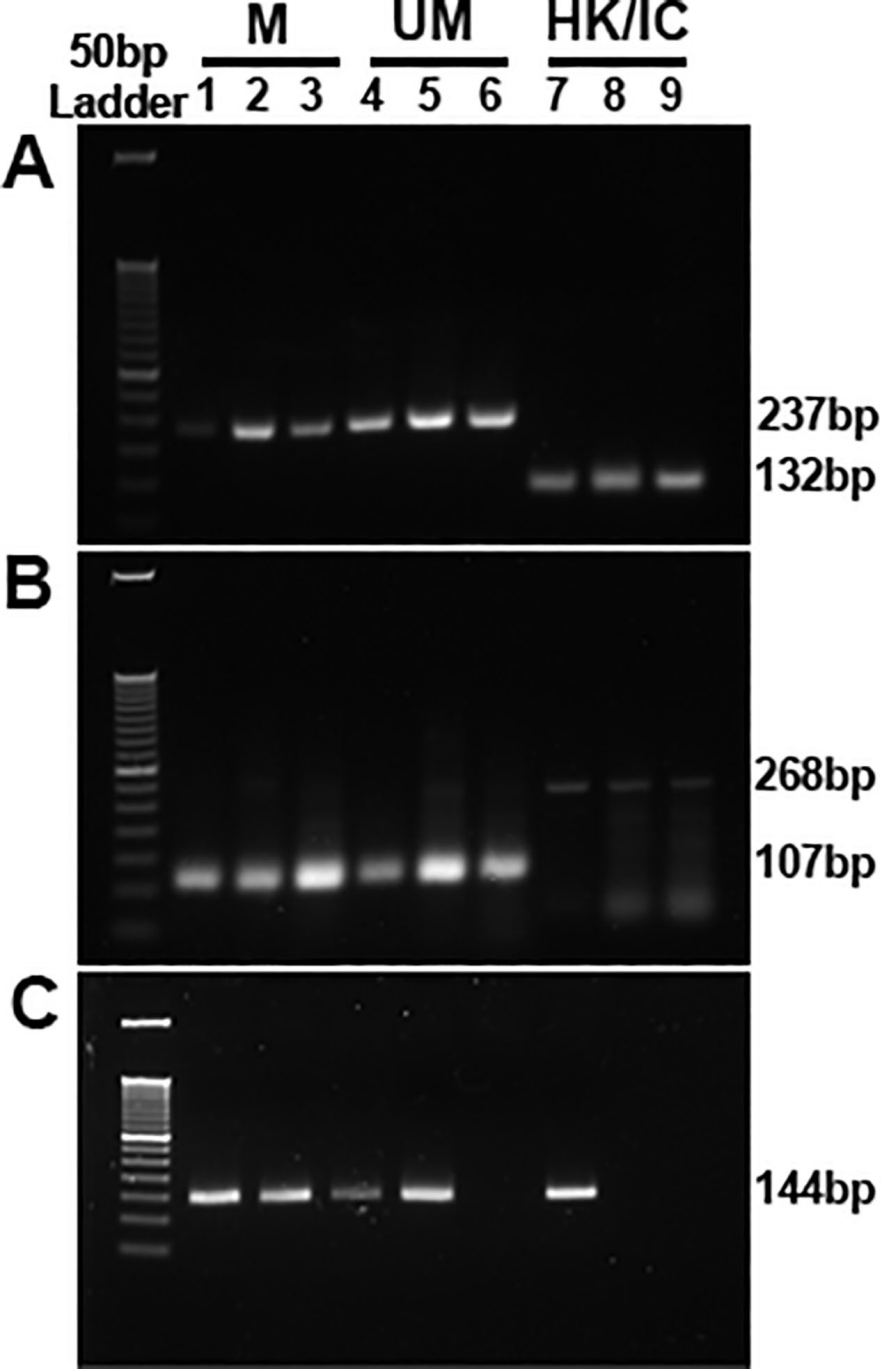
Representative agarose gel (3%) images showing effect of NaB treatment in CMF on gene promoter methylation of **(A)** *αSMA*, **(B)** *Col III*, and **(C)** *FSP1* genes as analyzed by methylation specific PCR. Lanes 1, 2, 3 represent methylated (M) promoter; lanes 4, 5, 6 represent unmethylated (UM) promoter; lanes 7, 8, 9 in panel A represent housekeeping (HK) gene (ACTB, beta actin) and lanes 7,8,9 in panel B represents internal control (IC) for methylation (DAPK, death associated protein kinase). Lanes 1,4,7: CSF; Lanes 2,5,8: CMF; and Lanes 3,6,9: CMF+NaB. NaB treatment altered gene promoter methylation of CMFs with hypermethylation of *αSMA* and *Col III*, while hypomethylation was noted in *FSP1* gene promoter corresponding with gene and protein expression levels as shown in Figs. 2 and 4, respectively. Untreated CSF cells were taken as control for determining the basal level of gene promoter methylation.

Figure 4 shows that the acquisition of fibroblastic morphology, changes in the mRNA, and protein expression in NaB-treated CMFs involves alterations in fibrotic gene promoter methylation. MSP analysis using primers specific for methylated (M; lanes 1–3) and unmethylated (UM; lanes 4–6) promoter regions of *αSMA*, *Col III* and *FSP1* genes, revealed hypermethylation of *αSMA* (Fig. 4A, lanes 3 and 6) and *Col III* (Fig. 4B, lanes 3 and 6) was concomitant with low gene expression noted in NaB-treated CMFs compared to the NaB-untreated CMFs (Figs. 2A, 2B). Furthermore, NaB-treated CMFs demonstrated hypomethylation of *FSP1* at gene promoter level (Fig. 4C, lanes 3 and 6) with corresponding increase in *FSP1* expression compared to NaB-untreated CMFs (Fig. 2D).

### NaB Induces Dedifferentiation in Corneal Myofibroblasts

**Figure 5.**
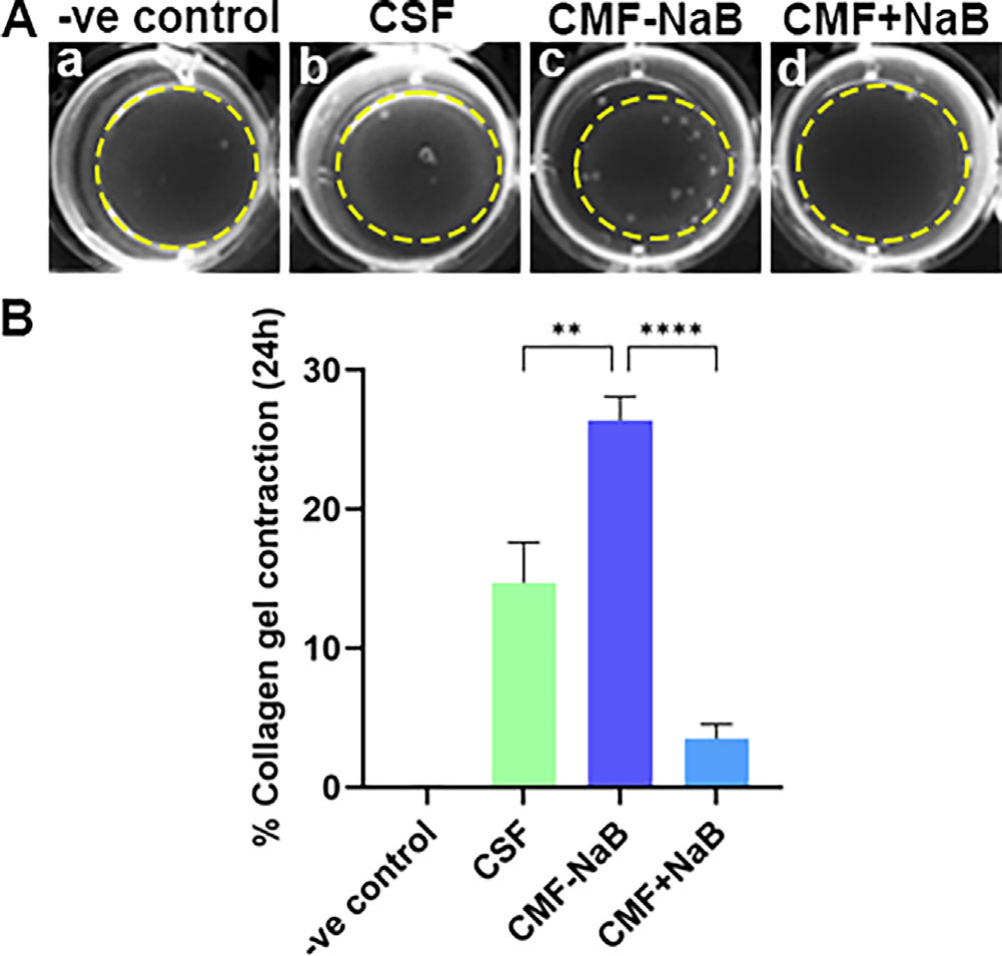
**(A)** Representative collagen gel contraction images, and **(B)** bar graph showing functional effect of NaB treatment in CMF contractility compared to untreated CSF. CSF+TGFβ1 (CMF) without NaB (*purple bar*) showed a significantly high contractility compared to unstimulated fibroblast (CSF, *green bar*; *p <* 0.01). NaB treatment significantly inhibited the contractility of CMF (*p <* 0.0001, *blue bar*) in collagen gel. Collagen gel without cells was taken as negative control for contraction; untreated CSF were taken to determine the basal level contraction of fibroblast.

The effects of NaB on CMF's contractility and dedifferentiation were evaluated by employing in vitro collagen gel contraction assay and in-gel immunostaining. Figure 5 shows the functional response of NaB on CMF's contractility in a tissue culture dish (Fig. 5A) and quantification of collagen gel contraction (Fig. 5B) at 24 hours. As evident from Figure 5, NaB-treated CMFs showed a significantly reduced collagen-gel contraction (3.5% ± 1%, *P* < 0.0001) compared to the NaB-untreated CMFs (26.4% ± 1.6%).

**Figure 6.**
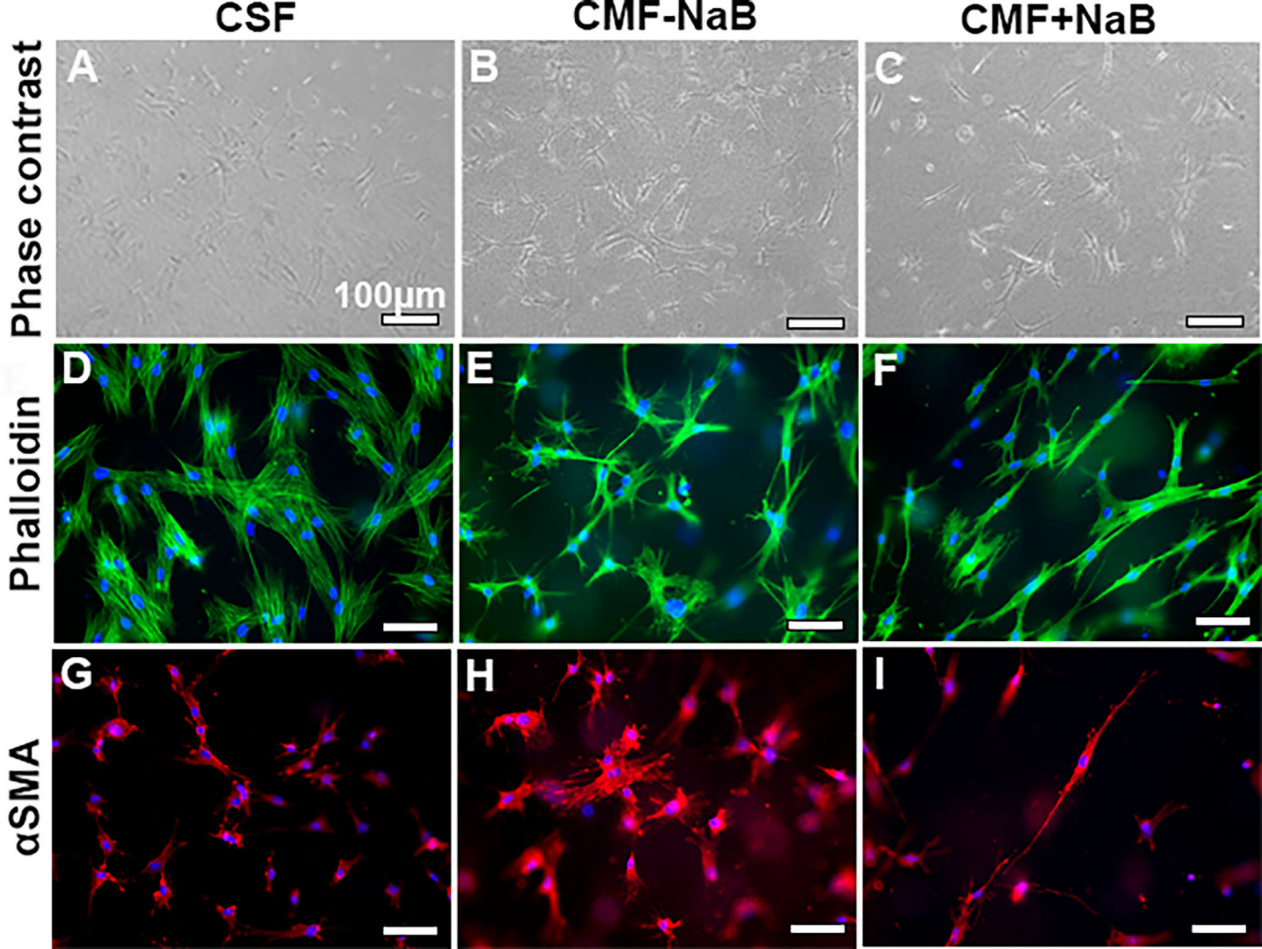
Representative collagen gel (**A**–**C**) phase contrast, and (**D**–**I**) in-gel immunofluorescence images showing functional effect of NaB treatment on expression of *αSMA* (stress fiber; **G**–**I**) responsible for contractile nature of myofibroblast along with morphological changes. NaB treatment reduced the expression of *αSMA* by 61.2% (1.85 ± 0.64 *vs*. 4.77 ± 0.02, *p* = 0.01) and contractility in CMF while expression of phalloidin (F-actin; 4.03 ± 0.77 vs. 4.72 ± 0.14; **D**–**F**), cytoskeletal protein, was akin to NaB-untreated CMFs.

The effect of NaB on CMF's dedifferentiation was investigated by in-gel double IF analysis using antibodies specific for phalloidin F-actin (cytoskeleton marker) and αSMA (myofibroblast marker). As apparent from Figure 6, NaB-treated CMFs showed 61.2% decrease in αSMA expression compared to the NaB-untreated CMFs (1.85% ± 0.64% vs. 4.77% ± 0.02%, *P* = 0.01; Fig. 6I vs. 6H) indicating the ability of NaB to inhibit CMF's contractility by promoting dedifferentiation. No appreciable change in the Phalloidin (F-actin) expression was observed in NaB-treated (4.03 ± 0.77) and -untreated CMFs (4.72 ± 0.14; Fig. 6F vs. 6E). The levels of F-actin (7.7 ± 0.01) and αSMA (2.16 ± 0.47) in baseline control of CSFs are presented in Figures 6D and 6G, respectively. Figures 6A–C show cellular morphology and density of CMFs at the time of seeding in collagen gels under phase contrast microscope. The contraction of collagen gel in NaB-treated vs. -untreated CMFs was also apparent at 48 and 72 hours (data not shown).

### NaB Reduces Corneal Fibrosis In Vivo in Rabbits

**Figure 7.**
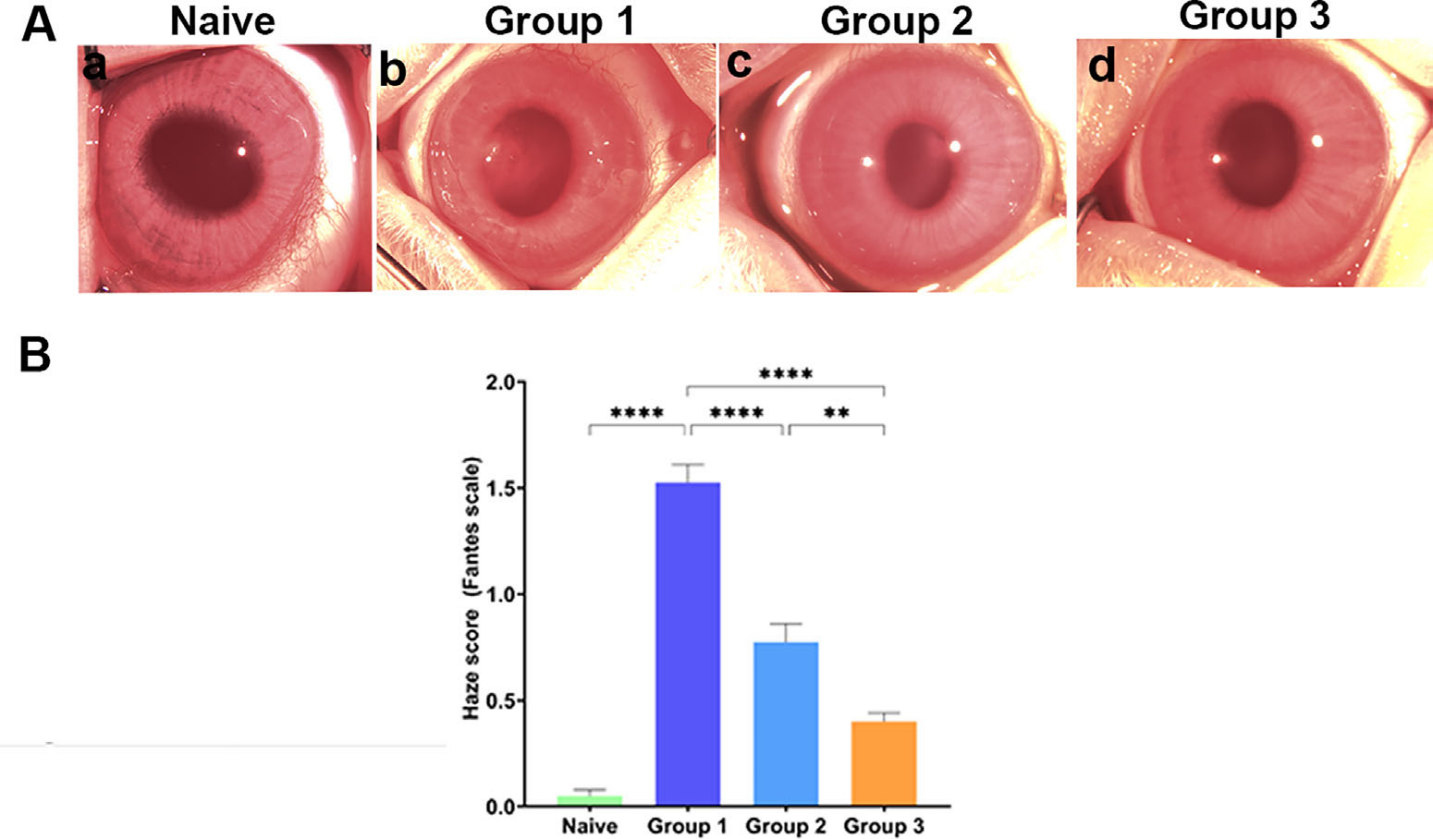
**(A)** Representative stereomicroscopic images showing the in vivo effect of NaB treatment on alkali injured rabbit cornea in the three groups at different time points. Group 1: alkali alone; Group 2: 5 mM NaB started one day after alkali injury twice daily for 7 days; Group 3: 5 mM NaB started 7 days after alkali injury twice daily for 7 days. Naïve, Group-1 and -3 imaged on D14; Group 2 imaged after 7 days of NaB treatment, respectively. **(B)** Bar graph showing a significant haze post-alkali injury (group1; *p <* 0.01). NaB treatment reduced the haze in group 2 and group 3 rabbits compared to group 1 (alkali alone).

Figure 7 presents functional response of NaB-induced CMF dedifferentiation in reducing corneal opacity in vivo in rabbits observed in clinical eye examinations with Stereomicroscope (Fig. 7A) and haze score (Fantes scale) quantification with slit-lamp microscope (Fig. 7B). As evident from corneal images, NaB-treated alkali-injured eyes showed significantly reduced corneal haze/opacity (Groups 2 and 3; *P* < 0.0001, respectively) compared to untreated alkali-injured eyes (Group 1). Also, the gross clinical eye exams after stopping NaB noted increased corneal transparency in eyes that received NaB 7 days after initial alkali-injury (Group 3, Fig. 7Ad) than the eyes that had NaB one day after injury (Group 2, Fig. 7Ac). As expected, alkali-injured untreated rabbit eyes (Group 1, Fig. 7Ab) showed markedly high corneal haze compared to the Naïve corneas (Fig. 7Aa). The opacity scores (Fantes scale) of Naïve and experimental eyes corroborated with clinical eye examination. This pilot study suggests that increased corneal transparency in vivo in Group 3 is due to dedifferentiation of unwanted CMFs by NaB via epigenetic reprograming.

**Figure 8.**
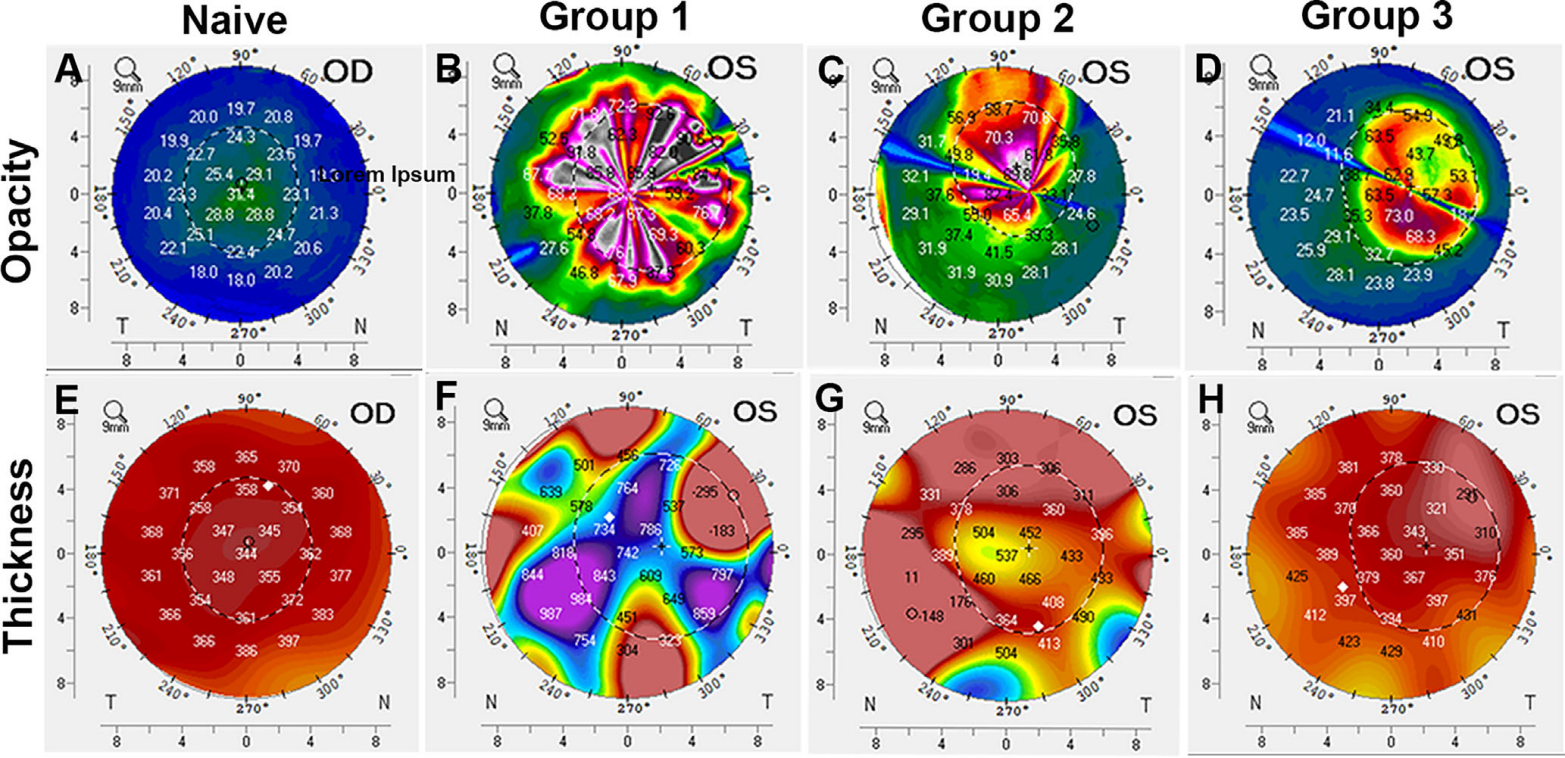
Representative Pentacam images showing the in vivo effect of NaB treatment on **(A–D)** corneal opacity, and **(E–H)** thickness in alkali injured rabbit cornea in the three groups compared to naive. NaB treatment reduced corneal haze (C; 40 ± 0.8, *p =* 0.04) and restored corneal thickness (G; 490 µm) in group 2 (5 mM NaB started one day after alkali injury twice daily for 7 days) rabbits. In group 3 (5 mM NaB started 7 days after alkali injury twice daily for 7 days) rabbits, haze (D; 33 ± 0.6, *p =* 0.02) and corneal thickness was further improved (H; 356 µm) compared to group 1 (B, 51.6 ± 3 haze; F, 704 µm thickness; alkali alone). The corneal opacity and thickness noted in naïve corneas was 21.6 ± 0.55 and 343 µm, respectively.

Figure 8 presents scores of corneal haze/opacity (A–D) and central corneal thickness (E–H) detected in Pentacam topography in a colored manner. A significantly decreased corneal opacity score was noted in NaB-treated alkali-injured Group 2 (Fig. 8C; 40 ± 0.8, *P* = 0.04) and Group-3 (Fig. 8D; 33 ± 0.6, *P* = 0.02) eyes compared to untreated alkali-injured Group 1 (Fig. 8B; 51.6 ± 3) eyes. The central corneal thickness scores revealed a pattern of clinical improvement by NaB akin to corneal opacity. The NaB-treated Group 2 (490 µm; Fig. 8G) and Group 3 (356 µm; Fig. 8H) eyes had a markedly reduced central corneal thickness compared to Group-1 (704 µm; Fig. 8F) eyes. The corneal thickness score of naïve corneas was 343 µm (Fig. 8E).

## Discussion

Corneal myofibroblasts are critical source of ECM and collagens in healing cornea but their accumulation and resistance to apoptosis lead to corneal fibrosis. Clearance of myofibroblasts via dedifferentiation has been shown to reverse pulmonary and cardiac fibrosis.^15^^,^^17^^,^^52^ The role of myofibroblasts (CMFs) in corneal tissue repair is well documented, but little is known about CMF dedifferentiation into precursor keratocytes/fibroblasts (CSFs). The present study demonstrates successful dedifferentiation of CMFs by NaB, a HDACi, to CSFs in human cornea in vitro and reduction of corneal fibrosis and attainment of transparency in rabbit eyes in vivo. Also, the reversal of CMFs to CSFs involves epigenetic reprograming in CMFs through inhibition of HDAC activity, hypermethylation of αSMA and Col-III, and hypomethylation of FSP1 gene promoters in human cornea in vitro.

Transdifferentiation of quiescent and transparent corneal keratocytes/fibroblasts into metabolically active opaque myofibroblasts (CMFs) post injury is a major mechanism to facilitate corneal repair.^1^^–^^3^ Failure in this precisely controlled mechanism leads to excessive CMF formation, and their persistence after wound closure results in corneal haze/opacity.^1^^–^^3^ Presently, there are no effective treatment strategies available to manage corneal fibrosis, drugs such as topical corticosteroids, mitomycin C (MMC), and topical losartan are being used in the clinic to manage corneal haze; however, these drugs show significant side effects.^53^^–^^55^ Corticosteroids (suppress inflammation) are often used for early haze, whereas MMC (inhibits fibroblast proliferation) is a highly effective preventive agent used during surgery after some procedures, although it has potential side effects.^53^^,^^54^ Recently, losartan (angiotensin II receptor blocker) has shown promise in reducing corneal haze.^55^^,^^56^ Although these drugs target inflammation, cellular proliferation, apoptosis, and TGFβ signaling, the present study explored an innovative approach that targets epigenetics to reverse abnormal gene expression associated with corneal fibrosis.

Literature shows that corneal fibrosis can be prevented by inhibiting proliferation of CSFs, blocking transdifferentiation of CSFs to CMFs, eliminating CMFs via apoptosis, or inducing degradation of fibrotic ECM.^1^^–^^5^ In 2001, Maltseva et al.^57^ reported CMFs and CSFs are alternative phenotypes after noticing morphological reversal in vitro when cultured in the presence of fibroblast growth factor and heparin and concluded these cells are not terminally differentiated cell types. Concomitant to earlier report, NaB treatment to CMFs switched myofibroblast phenotype to fibroblastic phenotype (Fig. 1), which was subsequently validated by qRT-PCR and IF studies. Decreased expression of myofibroblast indicators (αSMA, Col III, FN) and increased fibroblast (FSP1) indicator led to the development of novel working hypothesis that CMFs can sense needs of and stromal microenvironment in a healing cornea and has potential to reverse corneal fibrosis and restore transparency by undergoing dedifferentiation. The present study tested a novel postulate that NaB-induced DNA methylation changes lead to CMF dedifferentiation in corneal stroma and restore corneal transparency. To the best of our knowledge, it remains unknown until now whether CMFs can dedifferentiate into precursor CSFs in the corneal wound healing field.

The involvement of HDACs in TGFβ1 induced CMF formation and amelioration of fibrosis by HDACi such as vorinostat/SAHA and trichostatin A in rabbit cornea in vivo without major adverse effects are reported by us previously.^35^^–^^37^ Our earlier study revealed that anti-fibrotic effects of SAHA in cornea involve multiple mechanisms including the modulation of both canonical and non-canonical intracellular signaling pathways activated by TGFβ1 and MMP activity.^58^ One of the epigenetic mechanisms governing gene expression is changes in the pattern of gene promoter methylation, wherein hypermethylation is linked to gene repression while hypomethylation is linked to gene activation.^19^^,^^20^ Changes in the pattern of DNA methylation or chromatin accessibility (open or closed chromatin) particularly at gene promoter region due to histone modifications have been linked to fibrinogenesis.^19^^–^^25^ The present study uncovered that NaB effectively changes CMF's phenotype and fibrotic characteristics by altering HDAC activity and gene promoter methylation of fibrotic genes in human cornea in vitro model of fibrosis (Figs. 1^2^,^3^–4). The total HDAC activity was significantly reduced (Fig. 3) and gene promoters of αSMA and Col III (myofibroblast indicators) were hypermethylated whereas FSP1 (fibroblast indicator) was hypomethylated (Fig. 4). This data corroborated with our gene expression data finding of reduced αSMA, Col III and increased FSP1 expression (Figs. 1 and 2). These findings are consistent with literature reporting an attenuation of fibrosis in preclinical models of skin, peritoneum, lungs, and kidneys via epigenetic modifications.^59^^–^^69^

Myofibroblasts are highly contractile fibrogenic cell type that regulates ECM remodeling during fibrogenesis.^4^^–^^9^ The contractability of CMFs is typically governed by the αSMA exhibiting contractile stress fibers facilitating contraction, a key event during fibrosis. The CGA demonstrated significantly less contraction of collagen gel (Fig. 5) and αSMA expression (Fig. 6) in human cornea in vitro in NaB-treated CMFs compared to NaB-untreated CMFs which is consistent to hypothesis that NaB reduced CMF population. The in vivo applicability of this novel concept was verified by an in vivo pilot study with a standard alkali-injury rabbit model. Quantitative haze score of clinical slit-lamp (Fig. 7), and corneal opacity and thickness levels byPentacam imaging (Fig. 8) in NaB-treated versus NaB-untreated rabbit eyes showing significant attenuation of corneal haze/fibrosis and augmentation of corneal transparency are presumably due to reversal of CMFs.

Taken together, the results of our study, to the best of our knowledge, are the first description of the dedifferentiation of CMFs into precursor CSFs by NaB. The anti-fibrotic effects were demonstrated in a human cornea in vitro model and in vivo rabbit cornea alkali-injury model. Nevertheless, the study has a few limitations. A time-dependent experiment studied morphological changes in CMFs in the presence or absence of NaB (5 mM) at 24, 48, and 72 hours through dose- and time-dependent experiments (Supplementary Fig. S1). The highest change in CMF morphology by NaB was noted at 72 hours. Therefore we measured changes in total HDAC activity, DNA methylation, mRNA and protein expression of myofibroblast and fibroblast indicators at 72 hours only. Omission of timepoints before or after 72 hours could be seen as a potential limitation to understand time-dependent epigenetic changes occurring during CMF dedifferentiation. The relationship between the contractability of CMFs in CGA and αSMA and F-actin expression was investigated at 24, 48, and 72 hours, but only 24-hour data is presented (Figs. 5 and 6) despite finding remarkable collagen-gel contraction at 48 and 72 hours in alignment with αSMA and F-actin expression. This was due to a technical difficulty of high background immunofluorescence (αSMA and F-actin) noted at 48 and 72 hours, respectively. Another perceived limitation is pilot in vivo rabbit study used limited animals (*n* = 4) in each cohort. Furthermore, pilot rabbit study indicated that NaB may have a dual therapeutic functionality: preventing excessive formation of CMF by limiting transdifferentiation of CSFs to CMFs in early-stage of corneal repair post injury (Group-2) while promoting CMF dedifferentiation *via* epigenetic regulation (Group-3) in late-stage corneal repair. This study does not address this important concern due to funding constraints. Additional molecular/mechanistic in vivo studies are warranted to support or refute a notion dual therapeutic functionality of NaB and other HDACi. In essence, this study provides proof-of-concept that activation of targeted CMF dedifferentiation can lead to restoration of corneal transparency and consequently the vision in vivo.

In summary, this study provides a proof-of-concept that targeted CMF dedifferentiation in traumatized cornea by a suitable drug can lead to fibrosis reversal in vivo. Furthermore, it highlights an innovative approach to restore corneal transparency and vision by epigenetic modifier, NaB, which acts by reversing myofibroblast to less differentiated precursor cells. Additional in-depth investigations defining underlying mechanism and safety of NaB to ocular tissues are required to judge bench-to-bedside translational potential of the new therapeutic strategy.

## Supplementary Material

Supplement 1
