# Supplementary figures and images for: Epigenetic Reprogramming Via Sodium Butyrate Induces Corneal Myofibroblast Dedifferentiation In Vitro and Inhibits Fibrosis In Vivo
Source: Invest Ophthalmol Vis Sci. 2025 Nov 24;66(14):59. doi: 10.1167/iovs.66.14.59 (PMC12663874; doi:10.1167/iovs.66.14.59)

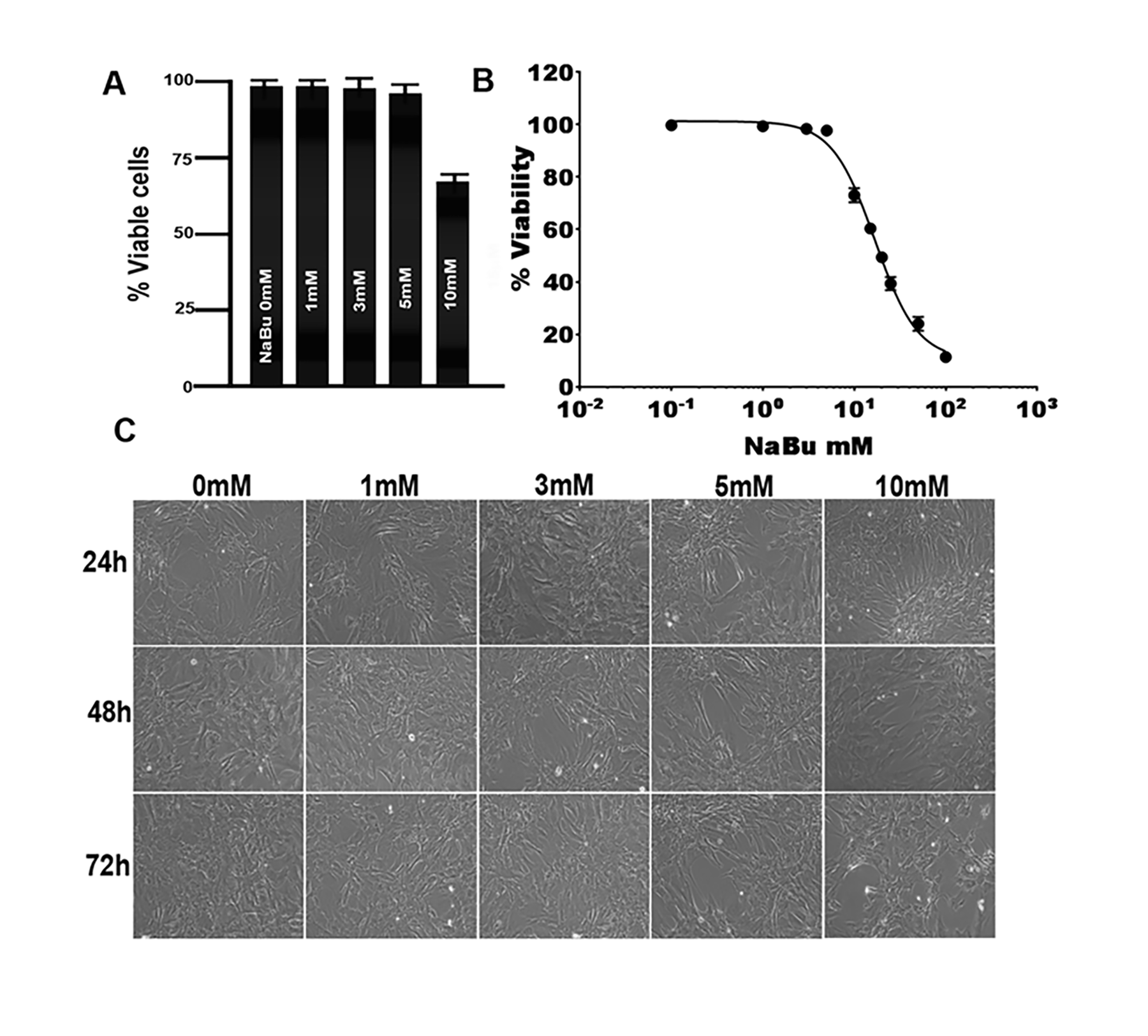

Supplement: Supplement 1 [file iovs-66-14-59_s001.tif]
